# Deep learning prediction of renal anomalies for prenatal ultrasound diagnosis

**DOI:** 10.1038/s41598-024-59248-4

**Published:** 2024-04-19

**Authors:** Olivier X. Miguel, Emily Kaczmarek, Inok Lee, Robin Ducharme, Alysha L. J. Dingwall-Harvey, Ruth Rennicks White, Brigitte Bonin, Richard I. Aviv, Steven Hawken, Christine M. Armour, Kevin Dick, Mark C. Walker

**Affiliations:** 1 Clinical Epidemiology Program, Ottawa Hospital Research Institute, Ottawa, Canada; 2 Children’s Hospital of Eastern Ontario Research Institute, Ottawa, Canada; 3 School of Epidemiology and Public Health, University of Ottawa, Ottawa, Canada; 4 ICES, Toronto, Canada; 5 Department of Pediatrics, University of Ottawa, Ottawa, Canada; 6 Prenatal Screening Ontario, Better Outcomes Registry and Network, Ottawa, Canada; 7 Department of Obstetrics and Gynecology, University of Ottawa, 501 Smyth Road, Ottawa, ON K1H-8L6 Canada; 8 International and Global Health Office, University of Ottawa, Ottawa, Canada; 9 BORN Ontario, Children’s Hospital of Eastern Ontario, Ottawa, Canada; 10 Department of Obstetrics, Gynecology and Newborn Care, The Ottawa Hospital, Ottawa, Canada; 11 Department of Radiology and Medical Imaging, University of Ottawa, Ottawa, Canada; 12 Department of Radiology and Medical Imaging, The Ottawa Hospital, Ottawa, Canada; 13 Neuroscience Program, Ottawa Hospital Research Institute, Ottawa, Canada

**Keywords:** Kidney anomaly diagnosis, Deep learning, Ultrasound imagery, Machine learning, Ultrasonography

## Abstract

Deep learning algorithms have demonstrated remarkable potential in clinical diagnostics, particularly in the field of medical imaging. In this study, we investigated the application of deep learning models in early detection of fetal kidney anomalies. To provide an enhanced interpretation of those models’ predictions, we proposed an adapted two-class representation and developed a multi-class model interpretation approach for problems with more than two labels and variable hierarchical grouping of labels. Additionally, we employed the explainable AI (XAI) visualization tools Grad-CAM and HiResCAM, to gain insights into model predictions and identify reasons for misclassifications. The study dataset consisted of 969 ultrasound images from unique patients; 646 control images and 323 cases of kidney anomalies, including 259 cases of unilateral urinary tract dilation and 64 cases of unilateral multicystic dysplastic kidney. The best performing model achieved a cross-validated area under the ROC curve of 91.28% ± 0.52%, with an overall accuracy of 84.03% ± 0.76%, sensitivity of 77.39% ± 1.99%, and specificity of 87.35% ± 1.28%. Our findings emphasize the potential of deep learning models in predicting kidney anomalies from limited prenatal ultrasound imagery. The proposed adaptations in model representation and interpretation represent a novel solution to multi-class prediction problems.

## Introduction

Deep learning (DL) is a machine learning methodology that has gained momentum in clinical diagnostics, particularly in medical imaging^1^. It utilizes artificial neural networks to process extensive datasets and identify important features that predict outcomes of interest^2^. Through the analysis of large clinical datasets, DL has demonstrated its ability to analyze vast amounts of data to provide invaluable prediction tools and clinical insights with the potential to enhance, and in some cases transform, healthcare delivery. This approach brings significant advantages, as DL model performance can be continuously improved as more data accrues. Notably, those models excel at pattern recognition, making them ideal for analyzing medical images. Convolutional neural networks (CNN), a specific DL architecture, has been identified as the method of choice for analyzing and interpreting medical images^3,4^.

Ultrasonography, more so than other imaging modalities such as X-ray, computed tomography (CT), and magnetic resonance imaging (MRI), is heavily performer- and interpreter-dependent, presenting a challenge to the DL models in clinical practice^5,6^. Since ultrasound (US) technology is a vital tool for assessing fetal anatomy, many researchers have found DL useful in prenatal US processes^6^. To date, most of the DL research on antenatal US has focused on fetal biometry and confirming normal structures, including the heart and brain^7^ . There have been only limited applications of DL in identifying structural anomalies thus far^6,7^.

Congenital anomalies of the kidneys and urinary tract (CAKUT) are relatively common, affecting 1 in 500 live births and comprising 20–30% of congenital fetal anomalies^8,9^. The kidney and urinary tract are vital organ systems that produce amniotic fluid and facilitate lung development^10^. Although various CAKUT can be detected during prenatal US exams, the false-positive rate is relatively high^11^. Therefore, more accurate diagnosis of CAKUT by prenatal US screening could improve fetal outcomes and reduce avoidable anxiety for expecting parents.

Previously, our research team successfully applied DL algorithms to the detection of cystic hygroma from fetal ultrasound images^12^. To further investigate the role of DL algorithms in medical imaging, we consider fetal kidney anomalies given their high incidence in the general population and the significant clinical impact of early detection. Conversely, the incidence of specific CAKUT subtypes such as multicystic dysplastic kidney (MCDK) and urinary tract dilation (UTD) is relatively low. The objective of this study was to determine the accuracy of a DL model in identifying CAKUT and establish the achievable class-specific discrimination of specific subtypes of CAKUT. We used several XAI explainability tools, including Gradient-weighted Class Activation Mapping (Grad-CAM) and High-Resolution Class Activation Mapping (HiResCAM)^12^ to facilitate the interpretation of our final model. This study makes key contributions: (1) the application of explainable deep learning for multi-class prediction of kidney anomalies and (2) an adapted two-class representation for a multi-class dataset through variable hierarchical grouping of labels to facilitate performance comparison with a binary classifier. We believe this work represents the first large-scale investigation of congenital kidney disorders in ultrasound images using deep learning with explainable AI.

## Data and methods

### Study setting and data acquisition

In this retrospective study, we included US images of singleton and twin pregnancies taken at a multi-site tertiary-care facility in Ontario, Canada between March 2014 and November 2021. This study was approved by the Ottawa Health Sciences Network Research Ethics Board (OHSN-REB). All study procedures were performed in accordance with the relevant guidelines and regulations. All images were captured using either a GE voluson™ V830 (916 images) or V730 (53 images) ultrasound system, by fully trained sonographers in obstetrics, and interpreted by maternal–fetal specialists. Images for both ‘normal’ instances and those reporting CAKUT were obtained between 18 and 24 weeks of gestational age (GA). This window was selected because, following full differentiation of the renal corticomedullary system, fetal renal structures are typically well developed at the 18th week of GA, and early detection (before 24 GA) of fetal urinary tract anomalies could independently predict poor postnatal renal outcome^11^. Further information on patient age and image dimensions is provided in Supplementary Table S1.

Images from two-dimensional (2D) transverse planes of fetal abdomens, measuring the renal pelvis anteroposterior diameter, were extracted from the institutional Picture Archiving and Communication System (PACS) and saved in Digital Imaging and Communications in Medicine (DICOM) format. Since we only analyzed 2D US images, only images of MCDK and UTD were included as part of the CAKUT ‘abnormal’ class classification. For clarity, we denote this class as ‘abnormal’ and not ‘CAKUT’ to make explicit that only a subset of CAKUT conditions are considered in this work. We considered 4 mm as the cut off value of UTD as per the 2014 UTD classification^11^. We collected multiple images from patients who underwent several US exams within the designated GA range. We excluded images that either did not have a 2D transverse kidney section for diagnosis or were not captured in the standard gray scale of US imagery.

### Data preprocessing

**Figure 1:**
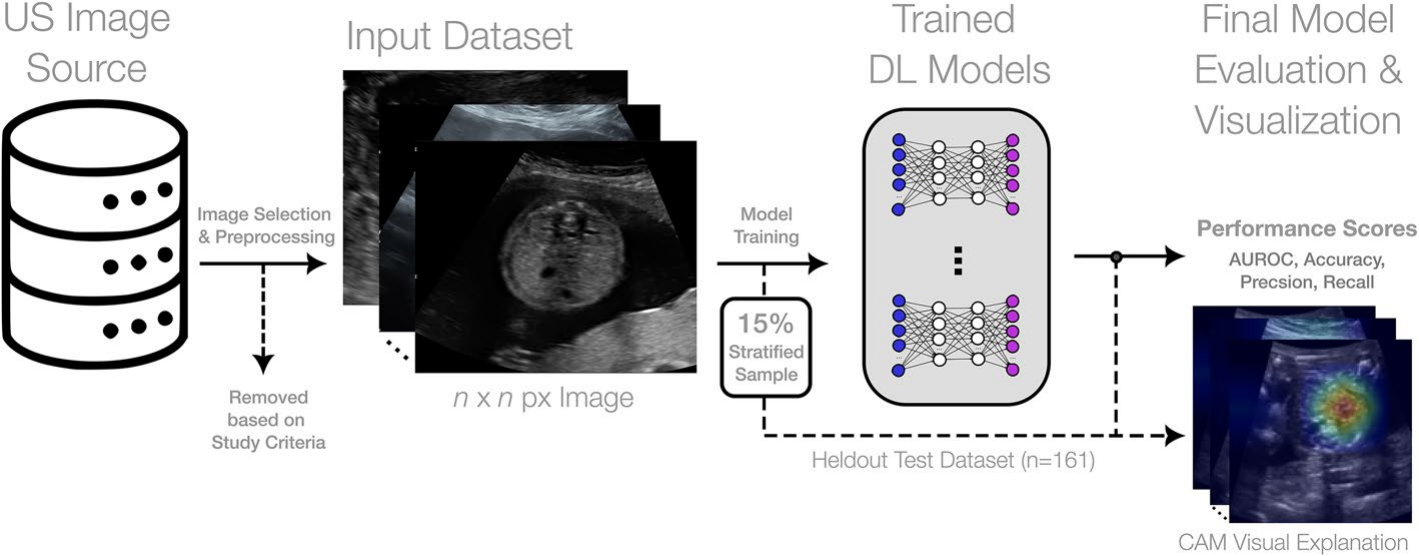
Conceptual overview of the model training and evaluation pipeline. From left to right, an original dataset of ultrasound images is preprocessed based on study inclusion criteria and transformed into a format for deep learning model trainings. A stratified sample of images is heldout exclusively for final model evaluation. The remaining training data is used to generate numerous DenseNet169 models with varying hyperparameters and modelling configurations. Through fourfold cross-validation and 5 independent repetitions, the best DenseNet169 model is selected based on validation performance and is evaluated on the heldout dataset. We additionally generate various visual explanation plots to investigate the image features leveraged by the model to inform its final prediction.

**Figure 2:**
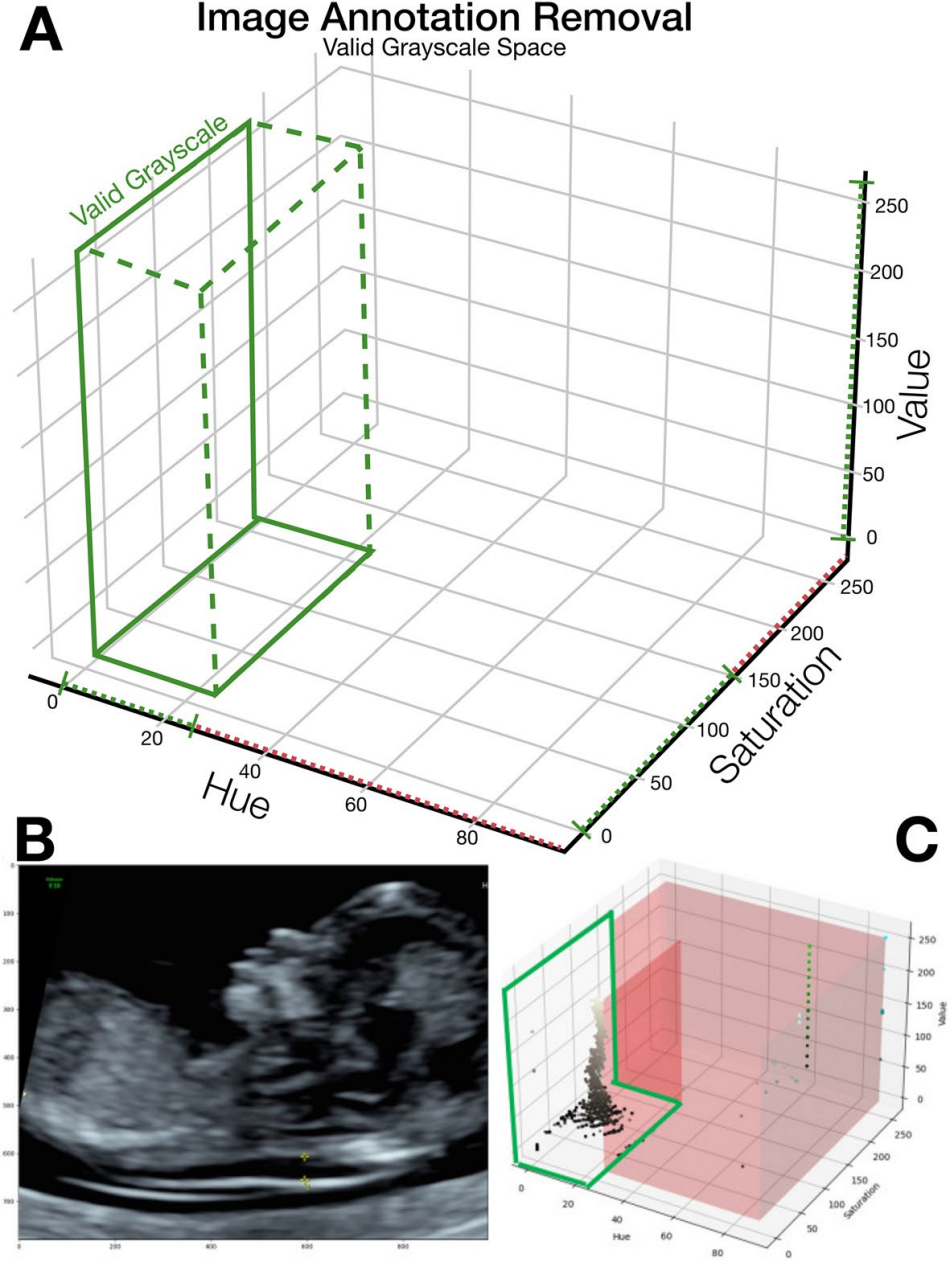
Image preprocessing for annotation removal. (**A**) depicts the valid grayscale HSV space applying the thresholds (0–27, 0–150, 0–255) for HSV respectively. (**B**,**C**) demonstrate the application of the preprocessing algorithm to an example ultrasound image; replicated from Walker et al.^12^.

Figure 1 depicts the conceptual overview of this study. The DICOM images used in the study required variable degrees of preprocessing prior to their use within the DL framework. A subset of images contained various coloured annotations such as calipers, text, icons, and profile traces. Another subset of images contained patient personal health information (PHI) that was visually present. Following the de-annotation and de-markup framework presented by our team previously^12^, we removed both coloured markup elements and all PHI to limit the introduction of bias and/or the leakage of class labels (Fig. 2). All images were verified following de-annotation and de-markup to ensure the quality of the images within the DL modelling dataset.

The resulting dataset of images spanned three classes: the ‘normal’ (*i.e.,* control group) class and two ‘abnormal’ classes comprised of the MCDK and UTD images. The set of normal class images were randomly sampled from the full collection of available control imagery, conditioned on matching the years from which the abnormal images were captured. We performed this stratified downsampling of all available normal images to achieve a relative class ratio of ~ 2:1 for normal:abnormal instances. This control group of instances were normal axial kidney images extracted from pregnancies without CAKUT.

### Model training framework and performance metrics

**Figure 3:**
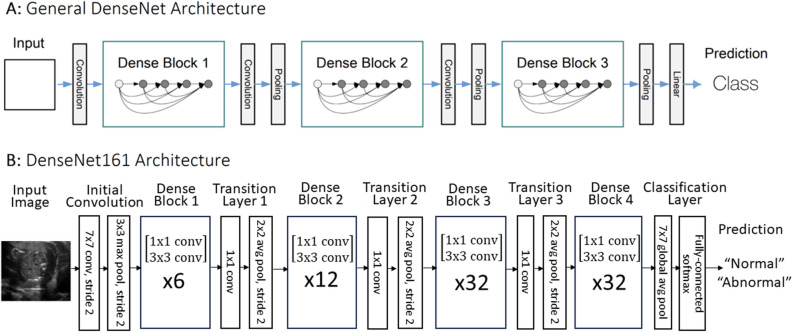
DenseNet model architecture.

Following the methodology used in our previous study focused on cystic hygroma^12^, we leveraged the Densely Connected Convolutional Networks (DenseNet) CNN model architecture to categorize images^13^. Specifically, we utilized the DenseNet169 PyTorch model, modifying the input layer to accommodate a variety of input image sizes (*e.g.*, $128 \times 128 \times 1$ or $256 \times 256 \times 1$ pixels). Depending on the specific experiment, the output layer was also adjusted to generate either two (binary) or three (multi-class) output values (Fig. 3). We used a weighted cross-entropy loss function with weights calculated using the inverse of the class frequency.

Throughout all experiments, the DenseNet169 models were trained from scratch over a variable number of epochs using fourfold cross-validation. Experiments were also repeated *k* = 5 times to compute accurate confidence intervals (CIs) of the reported performance and in plotting the Receiver Operating Characteristic (ROCs) and Precision-Recall (PR) curves. We use the normal approximation interval to compute 95% CIs on reported metrics from the independent folds and repetitions given the computationally expensive training of DL models. The performance metrics reported in this work include the area under the ROC curve (AUC), Accuracy, Sensitivity, Specificity, F1 Score and Precision; the latter five defined by the following equations:

1 $${\text{Accuracy}} = \frac{{{\text{TP}} + {\text{TN}}}}{{{\text{TP}} + {\text{TN}} + {\text{FP}} + {\text{FN}}}}{ }$$

2 $${\text{Sensitivity}} = \frac{{{\text{TP}}}}{{{\text{TP}} + {\text{FN}}}}$$

3 $${\text{Specificity}} = \frac{{{\text{TN}}}}{{{\text{TN}} + {\text{FP}}}}$$

4 $${\text{F}}1{\text{ Score}} = \frac{{{\text{TP}}}}{{{\text{TP}} + \frac{1}{2}\left( {{\text{FP}} + {\text{FN}}} \right)}}$$

5 $${\text{Precision}} = \frac{{{\text{TP}}}}{{{\text{TP}} + {\text{FP}}}}$$

where TP, TN, FP, FN denote the number of instances correctly predicted to be positive (true positive), the number of instances correctly predicted to be negative (true negative), the number of instances incorrectly predicted to be positive (false positive), and the number of instances incorrectly predicted to be negative (false negative), respectively.

### Hyperparameter tuning

To optimize the performance of our DenseNet169 models, we conducted hyperparameter tuning by varying three key parameters. Firstly, we resized the input images to three different dimensions: $128 \times 128 \times 1$, $256 \times 256 \times 1$, and $512 \times 512 \times 1$. This allowed us to investigate the influence of image resolution on both model performance and inference times. Secondly, we explored the impact of epoch number on performance by setting it to three different values: 100, 300, and 600. This enabled us to establish a baseline performance and determine whether longer training durations would lead to improved model accuracy. Lastly, we experimented with three different batch sizes: 32, 64, and 128. By varying the batch size, we aimed to evaluate the effect on model performance and the computational resources required for training. The adaptive setting of the positive weighting for abnormal classes was dependent on the prediction paradigm being considered, whether it was a two-class or three-class scenario. Through these systematic variations, we sought to identify the optimal combination of hyperparameters that would yield the best possible performance for our DenseNet169 models.

**Figure 4:**
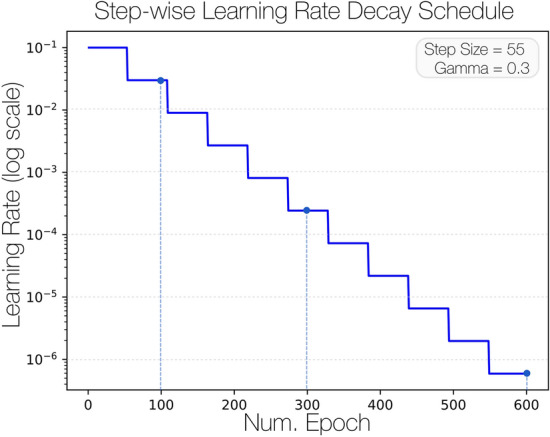
Learning rate decay schedule. The initial learning rate is set at 0.1 and follows a step-wise decay to enable the model to optimize performance. The learning rate values are represented with a log-scale to express the full range from $10^{ - 1}$ to $10^{ - 6}$ across the full span of training epochs.

Across all experiments, the learning rate was set according to a specific learning rate decay and schedule. This technique allows for the gradual reduction in the learning rate as training progresses to help the network converge more rapidly. We set the initial learning rate to 0.1 with a step size of 55 and a gamma of 0.3. In Fig. 4 we depict the varying learning rate step-wise decrease as a function of epoch number expressed with a log-scale for the maximal 600 epochs.

### Visual explanation of model predictions

To enhance the interpretability of our trained DenseNet models and provide visual context to the important features contributing to model predictions, we employed two methods from the emergent field of Explainable AI (XAI). The first, denoted the Grad-CAM method, is a widely recognized technique in the field of DL for visually explaining the behavior of algorithms^14^. Grad-CAM considers the gradients of the classification score relative to the final convolutional feature map, allowing for the attribution of influence to specific areas of an input image, highlighting those regions that exert the most influence on the classification score^14^. Notably, locations where the gradient is substantial correspond to regions where the final score heavily relies on the underlying data.

While the Grad-CAM method has been popularized within vision-based DL applications, recent investigations have brought to light a critical issue associated with its reliability^15^. It has been observed that Grad-CAM occasionally highlights regions within an image that were not utilized by the model for making predictions^15^. This study raises concerns regarding the trustworthiness of Grad-CAM as a model explanation method and an alternative proposed method, denoted HiResCAM, offers a promising solution by guaranteeing that it exclusively highlights locations that were genuinely utilized by the model. Conveniently, HiResCAM draws inspiration from Grad-CAM, simplifying model interpretability for those previously familiar with Grad-CAM.

In this work, we consider both Grad-CAM and HiResCAM as visual explanation methods to interpret the trained DenseNet169 model predictions. The complementary use of the two methods enables the intuitive and accurate interpretation of model predictions for end users. To generate the class activation maps (CAMs), we specify the *class_layer.relu* as the target layer; this layer represents the terminal rectified linear unit (ReLU) layer in the model. Given that the dimensions of the CAM are directly influenced by the size of the input image, we adaptively parameterize the CAM grid size to ensure that the resulting grid cells of the CAM are consistently $32 \times 32 \times 1$ pixels in size for a fair comparison across experiments. For example, an input image measuring $256 \times 256 \times 1$ uses a CAM grid size of $8 \times 8 \times 1$. Similarly, when the input image measures $128 \times 128 \times 1$, the resulting CAM grid is reduced to $4 \times 4 \times 1$. Here, we leverage the methods to confirm that the trained models indeed base their predicted outputs on regions of the image that clinicians would consider for the basis of their own classification and diagnosis.

### Adapted class representation due to limited dataset size

**Figure 5:**
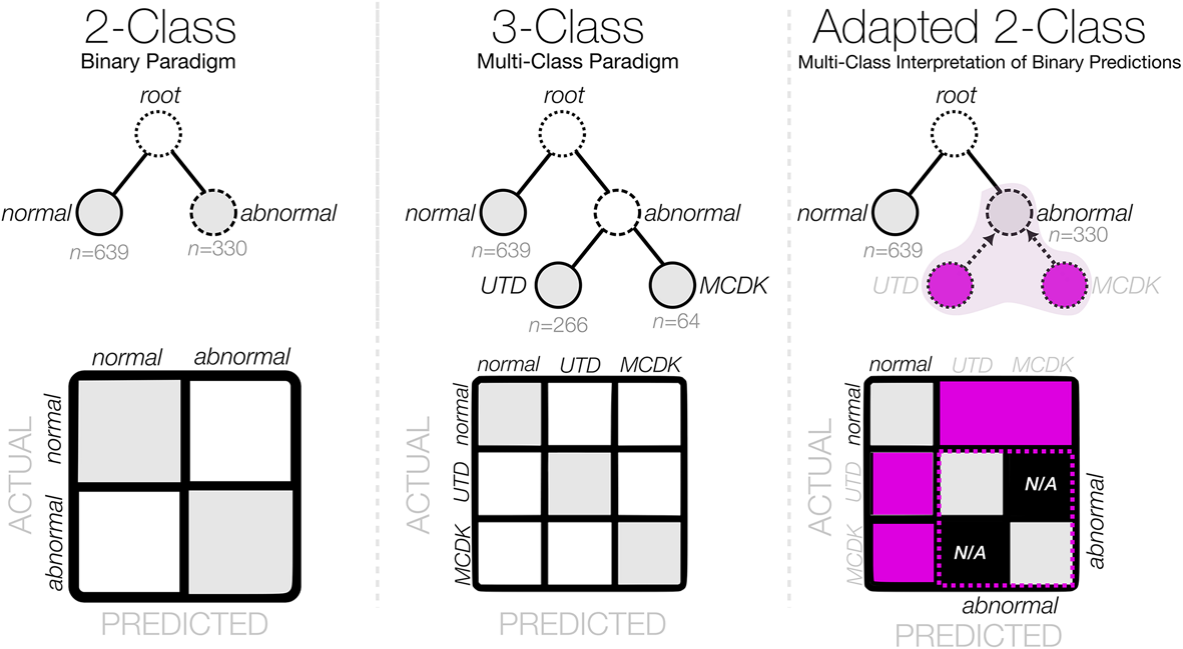
Conceptual overview of the prediction paradigms considered within this work. The 2-class and 3-class paradigms represent the standard prediction schemas for classifier modelling. The adapted 2-class confusion matrix contains both joined and N/A cells due to the inability to attribute a miss-classification between two labels within the same grouped metaclass.

In this work, we addressed the challenge of limited dataset size in the context of class representation for image data. Specifically, we aimed to investigate the classification of images into three distinct classes: normal, UTD, and MCDK. However, due to the limited number of available images in the CAKUT classes (UTD and MCDK with a total of only *n* = 259 and *n* = 64 images, respectively) we faced a significant imbalance in class distribution. To overcome this limitation, we adopted a pragmatic approach by grouping these images into a single "abnormal" metagroup, thereby mitigating the issue of data sparsity, and enabling a more balanced representation across the classes. By employing this adapted class representation strategy, we aimed to explore the impact of limited dataset size on classification performance, while also considering the practical constraints associated with acquiring a larger dataset for the UTD and MCDK classes (Fig. 5). To investigate the impact on model performance when formulating this as a 2-class problem (normal vs. abnormal) versus a 3-class problem (normal vs. UTD vs. MCDK), we trained an equivalent 3-class DenseNet model using the hyperparameters from the top-ranking 2-class models for a fair comparison.

This adapted 2-class representation also enabled the definition of an adapted multi-class model interpretation of the performance from binary classification, inviting a novel means of interpreting *k*-class predictions for a problem with > *k* labels and a variable grouping of hierarchical labels. The generalization of this concept and its impact on hierarchical prediction paradigms will be investigated as part of future work.

### Ethical approval

This study was reviewed and approved by the Ottawa Health Sciences Network Research Ethics Board (OHSN-REB #20210079). All methods were performed in accordance with the relevant institutional guidelines and regulations and in alignment with the Tri-Council Policy Statement: Ethical Conduct for Research Involving Humans (TCPS 2). This study involved images previously collected at the study centre, which were de-identified prior to model training and validation. Seeking participant consent was waived by the Ottawa Health Sciences Network Research Ethics Board as this retrospective study relied exclusively on secondary use of non-identifiable information. The data management and analysis for this study were conducted within the secure institutional network environment.

## Results

### Study sample

The complete image dataset included 969 ultrasound images from unique patients; 646 control images and 323 CAKUT (abnormal) cases, comprised of 259 UTD and 64 MCDK cases (Table 1). A total of 606 images were used for model training through fourfold cross-validation (~ 25% of the training withheld as a validation set per fold), and a final 161 images were used for final independent evaluation of the selected model.

**Table 1:** Partitioning of data across training, validation, and holdout test datasets.

	Overall, n (%)	Normal, n (%)	Abnormal	
			MCDK, n (%)	UTD, n (%)
Total	969 (100%)	646 (100%)	64 (100%)	259 (100%)
Training	606 (62.54%)	404 (62.54%)	40 (62.50%)	162 (62.55%)
Validation	202 (20.85%)	135 (20.90%)	13 (20.31%)	54 (20.85%)
Holdout test	161 (16.62%)	107 (16.56%)	11 (17.19%)	43 (16.60%)

*MCDK* Multi-cystic dysplastic kidney, *UTD* Urinary Tract Dilatation.

### CNN model validation and performance

**Figure 6:**
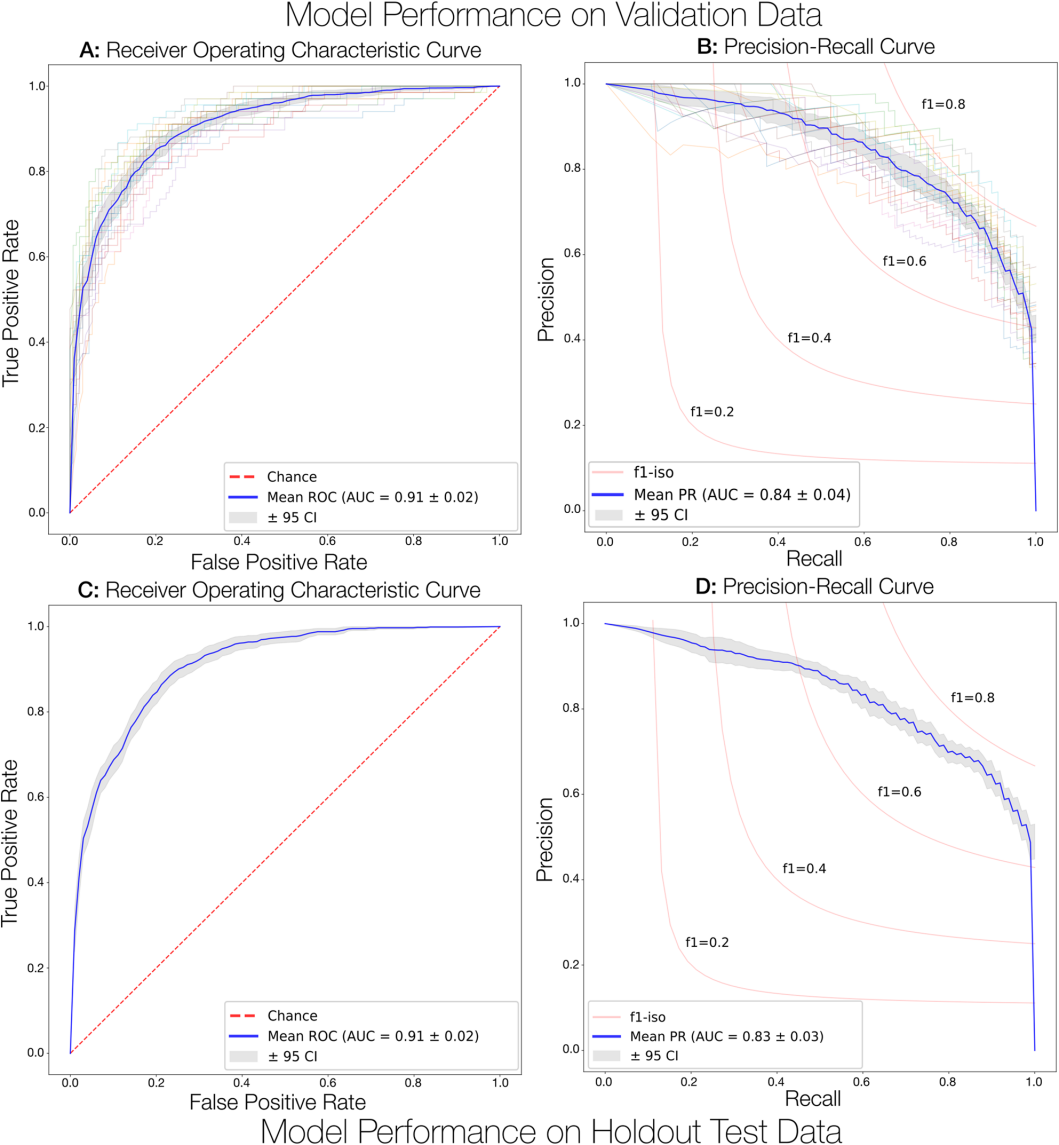
Model performance curves on the validation and holdout test dataset. (**A**,**B**) depict individual performance curves from five repetitions of four-fold cross-validation on the validation datasets. (**C**,**D**) summarize the final model performance on the heldout test dataset.

Table 2 summarizes the results from all model hyperparameter tuning experiments (sorted by descending AUC of the validation data), each reporting averaged performance metrics following fourfold cross-validation and 5 independent repetitions. The best performing model overall achieved an AUC of 91.28% ± 0.52%, a mean accuracy of 84.03% ± 0.76%, a mean sensitivity of 77.39% ± 1.99%, and a specificity of 87.35% ± 1.28% (Table 2). The top-performing model Receiver Operating Characteristic (ROC) and Precision-Recall (PR) curves are depicted in Fig. 6A,B.

**Table 2:** DenseNet161 model validation performance from 2-class hyperparameter tuning experiments following fourfold cross-validation and 5 independent repetitions.

Validation Rank	Hyperparameters			Validation Performance ($\mu \pm 95CI$)					
	Input Shape	Num. Epochs	Batch Size	AUC	Accuracy	F1 Score	Sensitivity/Recall	Specificity	Precision
1	128 × 128	300	32	0.913 ± 0.005	0.840 ± 0.008	0.763 ± 0.02	0.774 ± 0.020	0.874 ± 0.013	0.758 ± 0.023
2	128 × 128	300	64	0.900 ± 0.006	0.813 ± 0.007	0.750 ± 0.016	0.841 ± 0.014	0.800 ± 0.008	0.677 ± 0.015
3	256 × 256	600	64	0.894 ± 0.008	0.811 ± 0.011	0.737 ± 0.020	0.753 ± 0.022	0.854 ± 0.025	0.732 ± 0.036
4	128 × 128	300	64	0.892 ± 0.006	0.807 ± 0.008	0.732 ± 0.016	0.773 ± 0.016	0.831 ± 0.014	0.700 ± 0.025
5	256 × 256	300	32	0.888 ± 0.010	0.810 ± 0.011	0.732 ± 0.028	0.730 ± 0.023	0.868 ± 0.016	0.741 ± 0.033
6	128 × 128	100	64	0.884 ± 0.010	0.794 ± 0.011	0.732 ± 0.020	0.844 ± 0.016	0.769 ± 0.013	0.648 ± 0.021
7	128 × 128	300	128	0.870 ± 0.008	0.802 ± 0.012	0.717 ± 0.019	0.749 ± 0.017	0.827 ± 0.020	0.693 ± 0.032
8	256 × 256	300	64	0.866 ± 0.009	0.802 ± 0.009	0.700 ± 0.027	0.706 ± 0.032	0.849 ± 0.016	0.709 ± 0.030
9	256 × 256	300	64	0.866 ± 0.009	0.802 ± 0.009	0.700 ± 0.027	0.706 ± 0.032	0.849 ± 0.016	0.709 ± 0.030
10	512 × 512	600	64	0.862 ± 0.010	0.779 ± 0.023	0.682 ± 0.032	0.709 ± 0.042	0.813 ± 0.047	0.696 ± 0.057
11	256 × 256	300	128	0.830 ± 0.012	0.775 ± 0.009	0.657 ± 0.028	0.658 ± 0.035	0.834 ± 0.018	0.672 ± 0.027
12	256 × 256	300	128	0.830 ± 0.012	0.775 ± 0.009	0.657 ± 0.028	0.658 ± 0.035	0.834 ± 0.018	0.672 ± 0.027
13	512 × 512	300	64	0.824 ± 0.010	0.734 ± 0.011	0.661 ± 0.020	0.778 ± 0.017	0.712 ± 0.012	0.576 ± 0.020

For a fair comparison of model performance between the top-ranking 2-class model and an equivalent 3-class variant, Table 3 summarizes performance where we note a marked drop in performance in the 3-class prediction schema with an AUC of 75.46% ± 1.51%, a mean accuracy of 72.46% ± 1.18%, a mean sensitivity of 41.48% ± 2.51%, and a mean specificity of 86.05% ± 1.53%. Clearly, the limited availability of the instances for the UTD and MCDK classes made it challenging for the trained model to accurately differentiate these classes as compared to the binary problem formulation.

**Table 3:** DenseNet161 model comparison between top-ranking 2-class and equivalent 3-class variant.

Model	Hyperparameters			Validation Performance ($\mu \pm 95CI$)					
Binary vs. Multiclass	InputShape	Num. Epochs	Batch Size	AUC	Accuracy	F1 Score	Sensitivity/Recall	Specificity	Precision
2-Class	128 × 128	300	32	0.9128 ± 0.0052	0.8403 ± 0.0076	0.7630 ± 0.017	0.7739 ± 0.0199	0.8735 ± 0.0128	0.7580 ± 0.0231
3-Class	128 × 128	300	32	0.7546 ± 0.0151	0.7246 ± 0.0118	0.3820 ± 0.0436	0.4148 ± 0.0250	0.8605 ± 0.0153	0.3694 ± 0.0469

**Figure 7:**
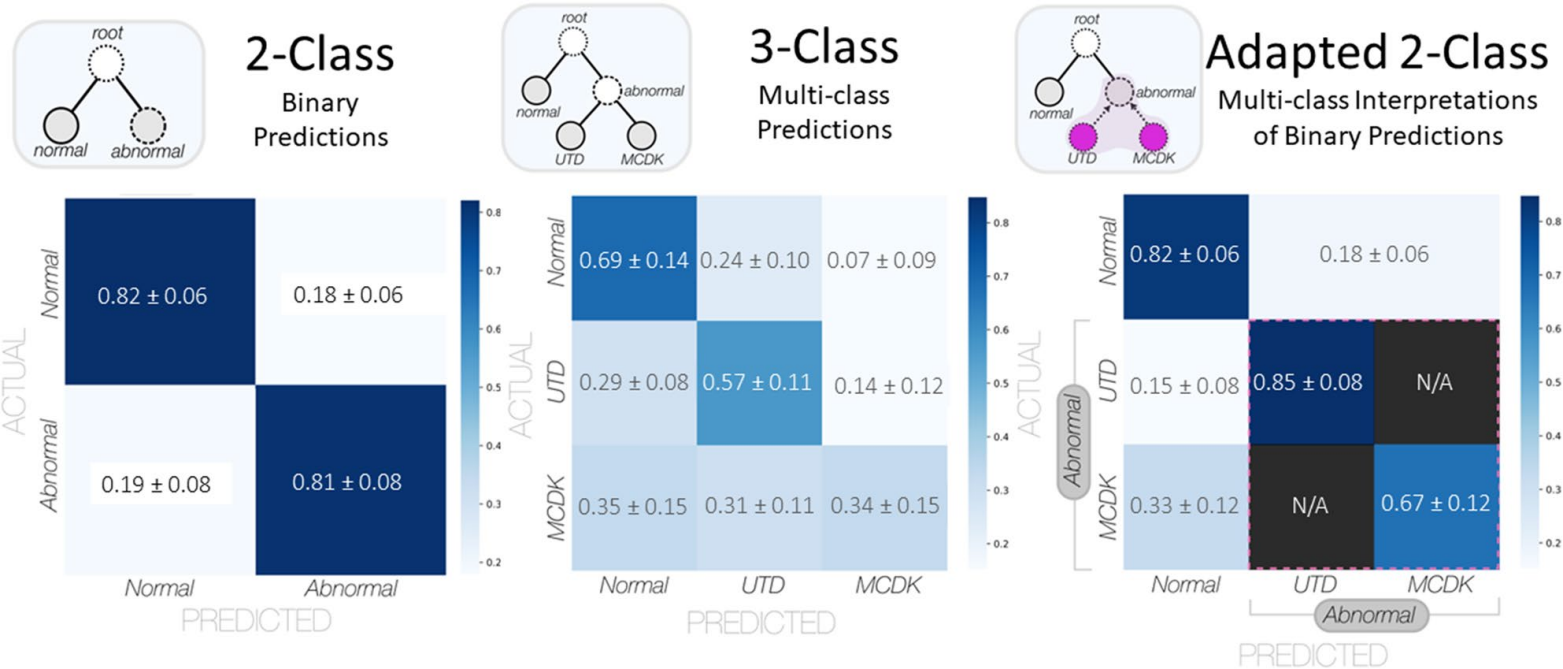
Comparison of confusion matrices from the 2-class paradigm, 3-class paradigm, and adapted 2-class paradigm. The left panel summarizes the performance of the final DenseNet169 model trained on binary labels and evaluated on the heldout test set (normalized $\mu \pm \sigma$). The middle panel summarizes the performance of an equivalent DenseNet169 model trained on multi-class labels and evaluated on the heldout test set (normalized $\mu \pm \sigma$). The right panel is an adapted confusion matrix representing the same performance of the binary classifier in predicting the outcome of the available three-class labels for comparison.

In Table 4, we report performance of the top-ranking model on an independently heldout test dataset (*n* = 161 test images). The model achieved a mean AUC of 90.71% ± 0.54%, a mean accuracy of 81.70% ± 0.88%, a mean sensitivity of 81.20% ± 2.40%, and a mean specificity of 82.06% ± 1.74%, each metric aligning well with the performance from the validation dataset, suggesting that this model generalizes well to other US imagery. In Fig. 6C,D, we visualize the ROC and PR curves when evaluating the final model on the heldout test dataset. The normalized confusion matrix summarizing the model performance on the heldout dataset is visualized in Fig. 7 along with an adapted 2-class representation to further interpret the binary model predictions based on the available 2-class labels. Interestingly, the binary classification model more reliably detects instances of UTD (85% ± 8%) than instances of MCDK (67% ± 12%). Supplementary Figs. S3, S4, and S5 illustrate the progression curves for the AUC, accuracy, and weighted cross-entropy loss, respectively. These figures highlight the performance of the top-ranking model across training epochs for both the validation and training datasets.

**Table 4:** Holdout test performance for the final model (top-ranking 2-class) ($\mu \pm 95CI$).

AUC	Accuracy	F1 Score	Sensitivity/recall	Specificity	Precision
0.9071 ± 0.0054	0.8170 ± 0.0088	0.7488 ± 0.0187	0.8120 ± 0.0240	0.8206 ± 0.0174	0.7007 ± 0.0231

### Visual explanation of model predictions

**Figure 8:**
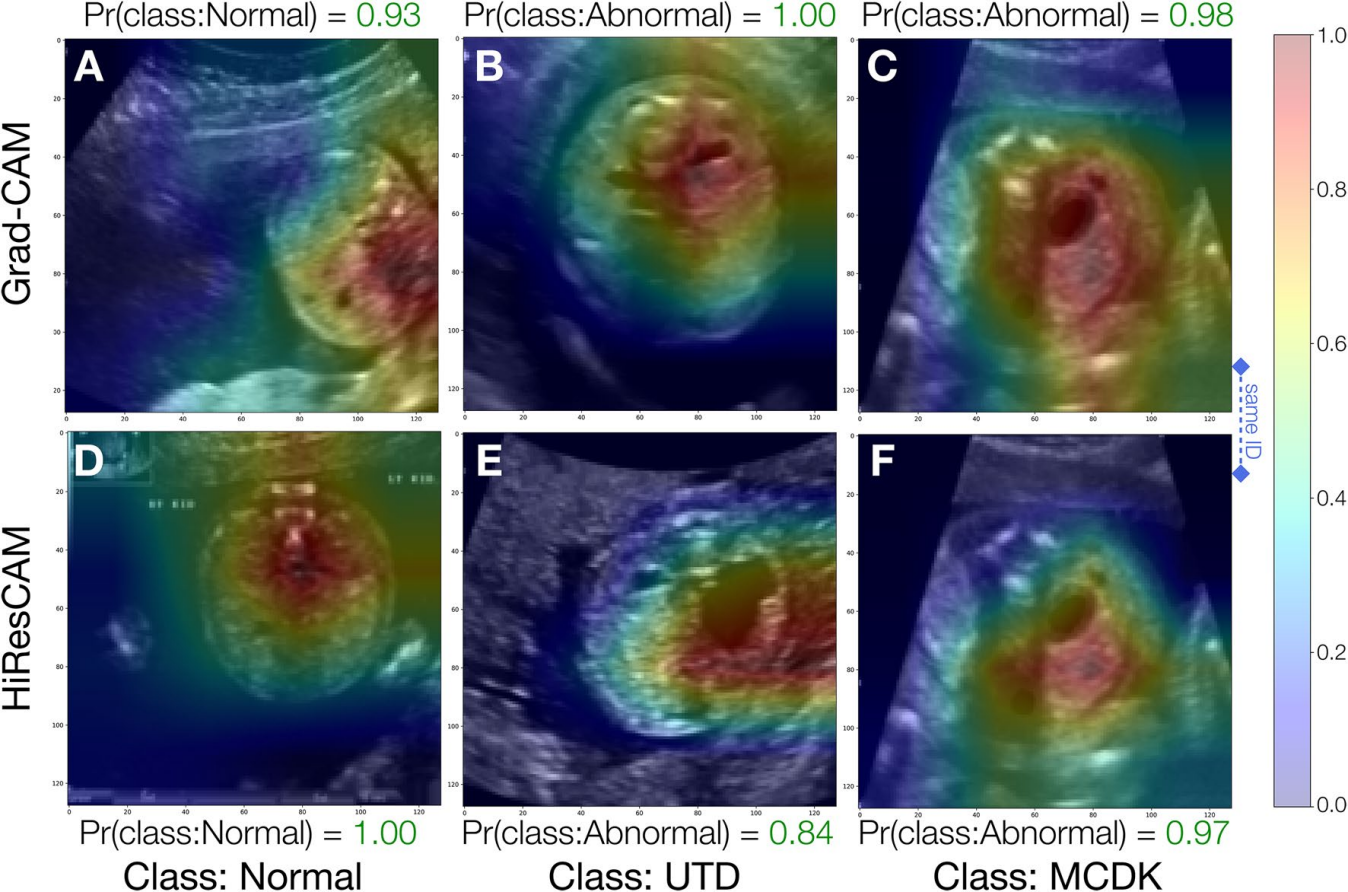
Sample visual explanations for successful model predictions. Panels (**A**–C) depict GradCAM activation maps whereas panels (**D**–**F**) depict HiResCAM activation maps. Panels in column (**C**,**F**) depict activation maps for the same patient ID to enable comparison of the activations maps of the two visual explanation methods for the same image. Pr(class:Normal) and Pr(class:Abnormal) are the probabilities for the Normal and Abnormal class respectively. The color bar units, ranging from 0.0 to 1.0, are unitless and denote the relative contribution of each pixel towards the model’s decision, where blue signifies low or negative importance and red signifies high importance.

We illustrate in Fig. 8 various examples of the activation maps from Grad-CAM and HiResCAM, obtained from the trained model when applied to instances from the holdout test set. Notably, across all classes and for both XAI methods, the model accurately focuses upon regions of the US image that would be clinically relevant for the diagnosis of CAKUT. In particular, we highlight comparisons between a Grad-CAM activation map and a HiResCAM activation map for the same image and note that the HiResCAM appears to hone in on more relevant regions of the image. Supplementary Fig. S1 presents additional cases where the model accurately targets clinically relevant regions.

### Model misclassification

**Figure 9:**
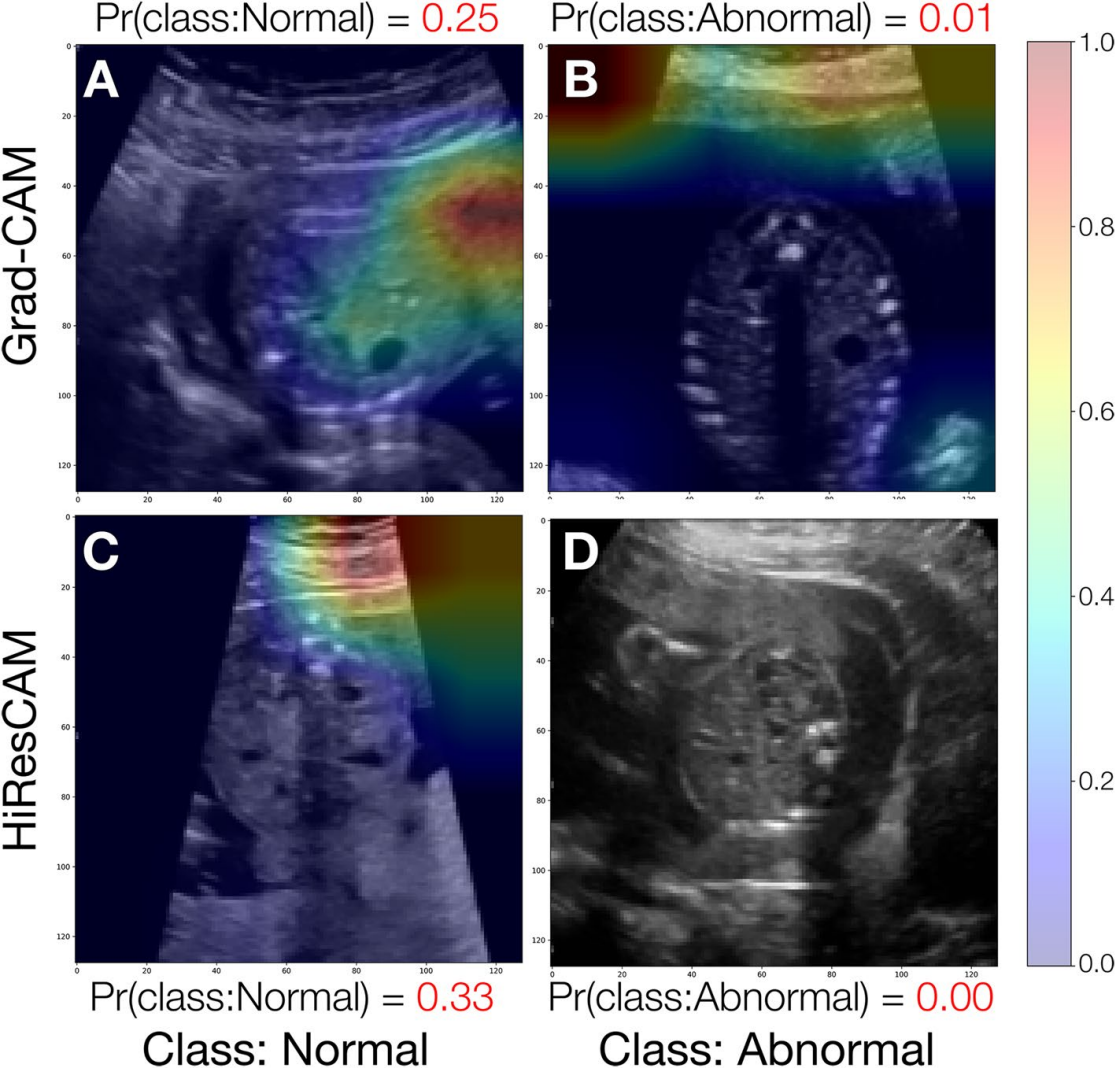
Sample of erroneous predictions and their visual explanation activation maps. Panels (**A**,**C**) depict a misclassified Normal kidney, panels (**B**,**D**) depict a misclassified UTD instance. Pr(class:Normal) and Pr(class:Abnormal) are the probabilities for the Normal and Abnormal class respectively. The color bar units, ranging from 0.0 to 1.0, are unitless and denote the relative contribution of each pixel towards the model’s decision, where blue signifies low or negative importance and red signifies high importance.

Two specific cases of misclassification by our model are highlighted in Fig. 9. For both cases the CAMs focus simultaneously upon an incorrect region (maternal abdominal wall) of the US image as well as external portions of the image. Supplementary Fig. S2 presents additional misclassified cases where the CAMs highlight regions which are not clinically relevant.

## Discussion

In this study, we demonstrate that DL algorithms can effectively make binary decisions, distinguishing between normal and abnormal conditions, based on retrospectively collected 2D fetal kidney US images. The top-performing model trained and evaluated within this work achieved an AUC around 90% and an accuracy greater than 80% on independently heldout test data, showing promise in its ability to generalize to independent US images containing CAKUT. A novel facet of this work is the flexible interpretation of multi-class performance from binary-type predictions. We generated a highly accurate 2-class DL model from which we could interpret 3-class labels (with the UTD and MCDK labels grouped into the ‘abnormal’ metaclass), thereby investigating what proportion of the abnormal labels were more reliably predicted, despite our binary problem formulation. Interestingly, the adapted 2-class confusion matrix suggested that the proportion of UTD instances can be more accurately predicted (85% ± 8%) than instances of MCDK (67% ± 12%). It is important to note that within the heldout test set, only 11 instances of MCDK were present as compared to the 43 UTD instance, suggesting that visually, with a greater number of available instances for training, the UTD images are more distinguishable from normal US images.

Significant advancements have been made in obstetric ultrasonography over the past four decades, enabling physicians to safely and effectively assess fetal conditions and accurately diagnose structural anomalies. However, despite improvements in US technology, ultrasonograms still heavily rely on the skills of the performer, which can contribute to under- or over-diagnosis, lead to medical malpractice concerns, and hinder accessibility in disadvantaged areas. It has been recognized that DL algorithms have the potential to serve as practical aids for performers of fetal ultrasonography, facilitating accurate image acquisition and diagnosis^16^. In a recent example, Xie et al*.* have reported the feasibility of utilizing AI models to diagnose fetal brain abnormalities^17^ and our prior research work has evidenced the use of DL models for the diagnosis of cystic hygroma from fetal US imagery^12^. While the detection of fetal congenital abnormalities represents a critical objective in fetal ultrasonography, there is a paucity of reports demonstrating the application of AI for CAKUT diagnosis and, of those reported examples, only limited transfer learning-based experiments exist^18–20^. To the best of our knowledge, this study represents the first large-scale initiative to investigate US images of CAKUT using a DL algorithm trained from scratch and leveraging XAI techniques to interpret model predictions.

There are several limitations and strengths to this study. First, this study only leverages data collected from a single center and thus, the sample size for developing and validating DL models is relatively small. As part of our mitigation strategy, we implemented the use of *k*-fold cross validation with numerous repetitions, and final model evaluation on a fully heldout test dataset. This approach is widely recognized as effective in mitigating the challenges associated with small datasets^21^. A strength of this work is our investigation of the hierarchical grouping of CAKUT through a complimentary binary (‘normal’ vs. ‘abnormal’) and multi-class (‘normal’ vs. ‘MCDK’ vs. ‘UTD’) experimental design, enabling a fair comparison between prediction paradigms with varying number of classes. This introduces a novel concept on how variable grouping of instances within classes or meta-classes can mitigate a lack of data within any one class, and explores the impact on model performance; consequently, this concept could be extended to other data representation hierarchies (including unsupervised learning where arbitrary class labels may be discarded in favor of embedding similarity).

Another limitation of this study is the lack of standardized data acquisition process for fetal ultrasound images, which raises concerns about data quality and consistency between normal and abnormal images. Within a retrospective study, we are limited by the quality and consistency of the data available to train a DL model and understandably, the DL model performance is dependent on both the quality and quantity of the imagery available to train and evaluate it. While the versatile comprehension of AI through emergent model explainability methods (*e.g.*, GradCAM & HiResCAM) can potentially aid in the analysis of these diverse data, the absence of a standardized method of data acquisition introduces risks. Furthermore, clinicians may not have a comprehensive understanding of the impact of data quality and consistency on model training, which increases the likelihood of introducing bias or class leakage into the dataset. This potential data leakage compromises the reliability and generalizability of the results obtained from the study. Therefore, it is essential to acknowledge the limitations stemming from the method of data acquisition and consider possible implications on the accuracy and robustness of the models.

The utilization of DL in ultrasonography has the potential to mitigate risks associated with the inherent nature of this imaging technique. Ultrasonography is heavily reliant on the operator, resulting in images that lack reproducibility and introduce subjectivity during acquisition and interpretation^22^. This dependence on the operator can lead to inconsistencies in image quality and diagnostic outcomes, posing challenges for physicians and limiting the broader utilization of ultrasound in medically underserved regions^22^.

Although DL-based models have demonstrated remarkable success across various domains in recent years, it is important to recognize that these models are not infallible, and they may occasionally produce erroneous predictions despite achieving high performance metrics. Several factors could contribute to the observed erroneous predictions. One possible explanation lies in the limitations of the training data itself. Despite efforts to curate high-quality and representative datasets, the presence of biased or noisy data can adversely affect the model's ability to generalize accurately. Input noise is a challenge in ultrasound imaging that affects the image features such as contrast and resolution. This is an area of active research, and many discussions exist on how to reduce noise while preserving the image quality and information^23–26^. Additionally, DL models are highly complex and often consist of numerous interconnected layers, making it challenging to interpret their decision-making process and pinpoint the source of errors. To better interpret the predictions of such a model, it is worth considering more intuitive model visualization techniques such as the recently proposed CAManim method^27^. Furthermore, DL models rely heavily on the optimization of loss functions during training, which may lead to overfitting or the exploitation of statistical patterns that do not truly generalize to real-world scenarios (*e.g.,* the regular occurrence of blackout regions from US scans). This can result in models that perform well at the task they were trained on, but fail when applied to unseen or ambiguous inputs, leading to unexpected and erroneous outputs. Our observations highlight the need for further research and development to enhance the reliability and robustness of DL models, particularly in critical domains where erroneous predictions can have severe consequences. With sufficient safeguards and human-in-the-loop review, we may improve our understanding of the underlying causes of these errors and strive towards increasingly trustworthy and accurate DL-based models that can be effectively deployed in real-world applications.

Future directions in evaluating the use of DL models in fetal US diagnostics should include prospective studies conducted with well-defined data collection protocols, established in consultation with a panel of both AI and subject-matter experts, who collectively possess a deep understanding of the clinical challenges as well as the impact of data quality and consistency on model training and evaluation. Data collection protocols can help mitigate the risks of data leakage, bias, and class leakage, and can ensure that the collected images are representative, consistent, and of high quality, leading to a more reliable and generalizable dataset. Such a protocol may also ensure that multi-institutional data collection remain compatible. The inclusion of AI experts in the development of the protocol will also enhance the understanding of the specific requirements and challenges associated with training machine learning models for fetal ultrasound diagnosis. Consequently, a prospective study with a robust data collection protocol will contribute to the advancement of accurate and trustworthy AI-based diagnostic tools in this domain.

## Conclusion

This study demonstrates the potential of DL algorithms in clinical diagnostics, specifically in the early detection of fetal kidney anomalies, using limited ultrasound imagery. This outcome encourages further development of DL algorithms capable of prospectively evaluating fetal US images in a multi-classification prediction schema. The potential establishment of such a DL system holds promise for the integration of AI-assisted prenatal US diagnostic systems in clinical settings, offering benefits to both patients and clinicians. By leveraging this technology, prenatal US examinations may be enhanced, leading to improved accuracy and efficiency in the diagnosis of fetal conditions, and ultimately, to improved patient care. The integration of XAI techniques strengthens our understanding of the model's decision-making process, enabling identification of areas for improvement, and fostering trust in deep learning predictions within clinical settings.

### Supplementary Information

Supplementary Information.

## Data Availability

The original image files are not publicly available due to their containing information that could compromise the privacy of research participants. The datasets generated and analyzed for this study are available from the corresponding author (MCW), upon reasonable request.
