# Supplementary material for: Deep learning prediction of renal anomalies for prenatal ultrasound diagnosis
Source: Sci Rep. 2024 Apr 19;14:9013. doi: 10.1038/s41598-024-59248-4 (PMC11031588; doi:10.1038/s41598-024-59248-4)
Supplement: Supplementary file 1 — Supplementary Information. [file 41598_2024_59248_MOESM1_ESM.docx]

**Supplementary Materials**

**Deep Learning Prediction of Renal Anomalies for Prenatal Ultrasound Diagnosis**

Olivier X. Miguel^1^, Emily Kaczmarek^2^, Inok Lee^1^, Robin Ducharme^1^, Alysha L.J. Dingwall-Harvey^1,2^, Ruth Rennicks White^1,7^, Brigitte Bonin ^7,10^ , Richard I. Aviv^11,12,13^, Steven Hawken^1,2,3,4^, Christine M. Armour^2,5,6^, Kevin Dick^2^*,* Mark C. Walker^1,2,3,5,7,8,9,10^

1. Clinical Epidemiology Program, Ottawa Hospital Research Institute, Ottawa, Canada
2. Children’s Hospital of Eastern Ontario Research Institute, Ottawa, Canada
3. School of Epidemiology and Public Health, University of Ottawa, Ottawa, Canada
4. ICES, Toronto, Canada
5. Department of Pediatrics, University of Ottawa, Ottawa, Canada
6. Prenatal Screening Ontario, Better Outcomes Registry & Network, Ottawa, Canada
7. Department of Obstetrics and Gynecology, University of Ottawa, Ottawa, Canada
8. International and Global Health Office, University of Ottawa, Ottawa, Canada
9. BORN Ontario, Children’s Hospital of Eastern Ontario, Ottawa, Canada
10. Department of Obstetrics, Gynecology & Newborn Care, The Ottawa Hospital, Ottawa, Canada
11. Department of Radiology and Medical Imaging, University of Ottawa, Ottawa, Canada,
12. Department of Radiology and Medical Imaging, The Ottawa Hospital, Ottawa, Canada,
13. Neuroscience Program, Ottawa Hospital Research Institute, Ottawa, Canada

This document contains supporting materials, tables and figures.

**Table S1** Patient Age and Image Sizes.

|  | **Count** | **Mean** | **Std** | **Min** | **5%** | **25%** | **50%** | **75%** | **95%** | **Max** |
| --- | --- | --- | --- | --- | --- | --- | --- | --- | --- | --- |
| **Patient Age** | 968 | 36.83 | 5.56 | 21 | 28 | 33 | 37 | 41 | 46 | 55 |
| **Width** | 969 | 851.01 | 149.45 | 629 | 629 | 804 | 804 | 804 | 965 | 1433 |
| **Height** | 969 | 680.84 | 60.69 | 528 | 528 | 663 | 663 | 663 | 780 | 780 |

**Supplemental Figures**


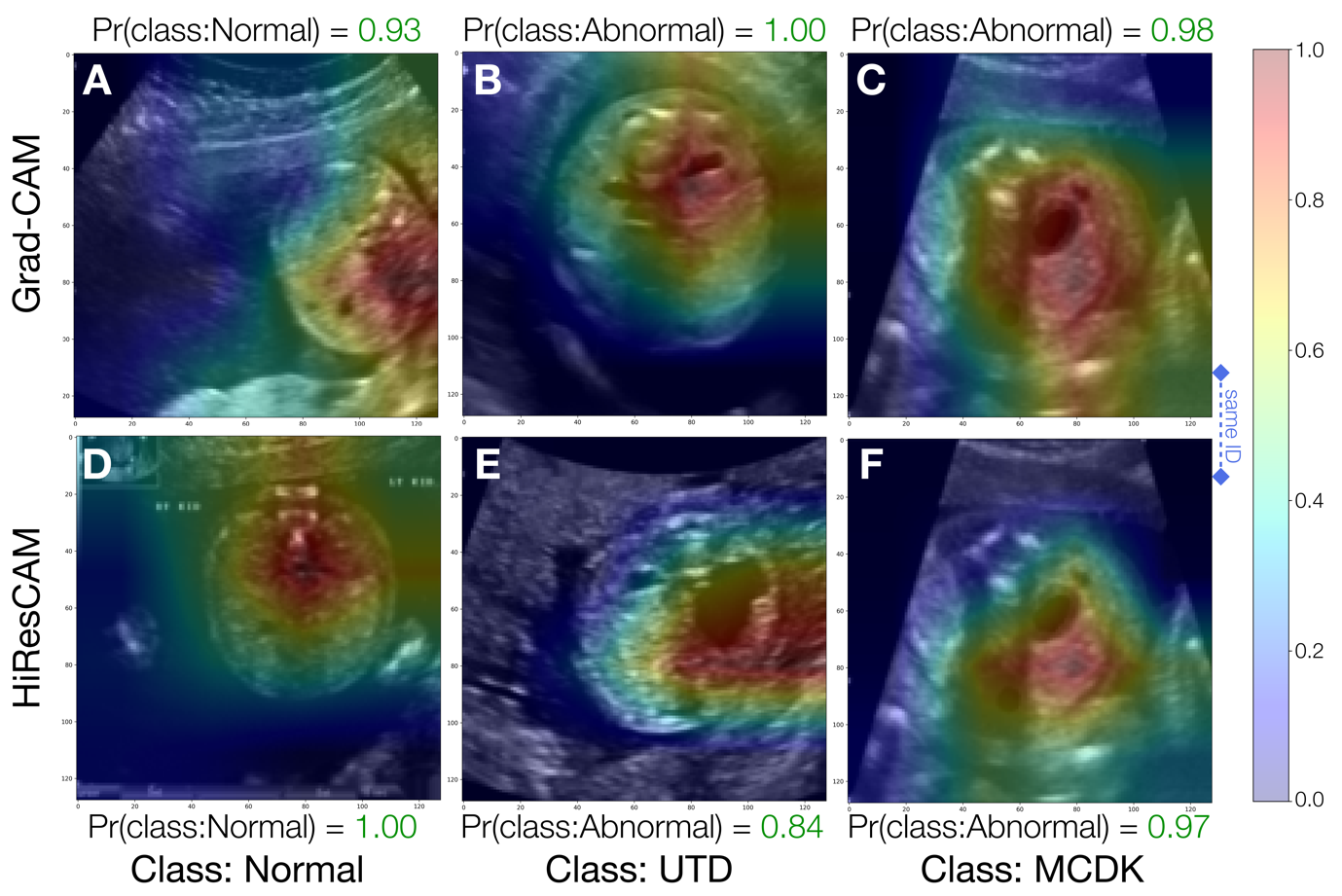


**Figure S1** **Sample Visual Explanations for Successful Model Predictions.** Panels A-C depict GradCAM activation maps whereas panels D-E depict HiResCAM activation maps. Panels C & F depict activation maps for the same patient ID to enable comparison of the activations maps of the two visual explanation methods for the same image. Pr(class:Normal) and Pr(class:Abnormal) are the probabilities for the Normal and Abnormal class respectively. The color bar units, ranging from 0.0 to 1.0, are unitless and denote the relative contribution of each pixel towards the model’s decision, where blue signifies low or negative importance and red signifies high importance.


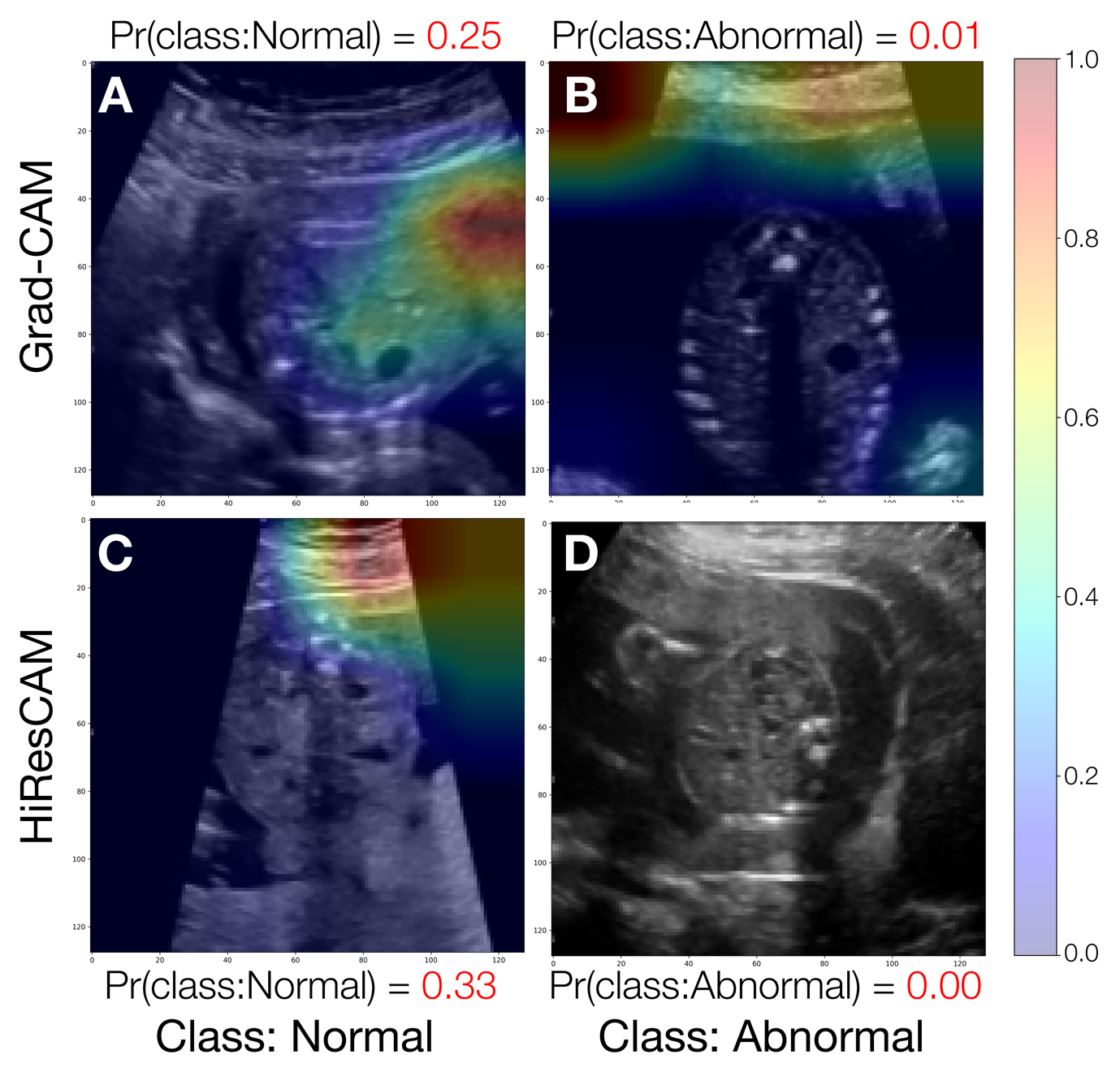


**Figure S2 Sample of Erroneous Predictions and their Visual Explanation Activation Maps.**

Four specific cases of misclassification by our model are highlighted in this Figure: the Grad-CAM activation map in A focuses upon an irrelevant portion of the US image; the Grad-CAM activation map in B appears to focus upon a region outside the US scan altogether (the maternal abdominal wall); the HiResCAM activation map in C focuses simultaneously upon an incorrect region (maternal abdominal wall) of the US image as well as external portions of the image; and panel D depicts a lack of activation map altogether. A lack of heatmap can occur when the model fails to identify any discriminative regions in the image to support its prediction.

Panels A & C depict misclassified Normal kidneys, panel B depicts a misclassified UTD instance, and panel D depicts a misclassified MCDK instance. Pr(class:Normal) and Pr(class:Abnormal) are the probabilities for the Normal and Abnormal class respectively. The color bar units, ranging from 0.0 to 1.0, are unitless and denote the relative contribution of each pixel towards the model’s decision, where blue signifies low or negative importance and red signifies high importance.


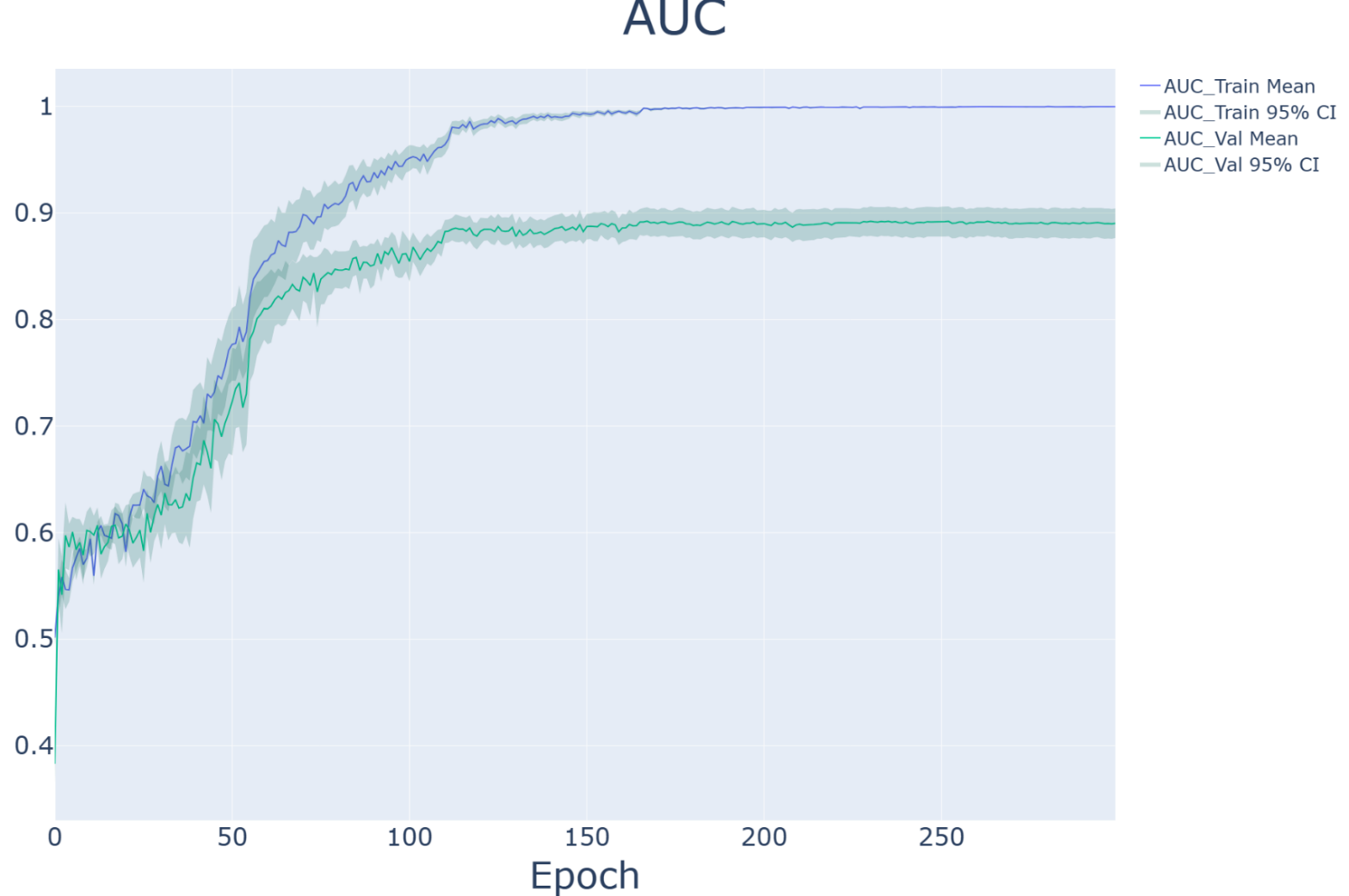


**Figure S3 Area under the ROC curve (AUC).** AUC on the training and validation sets over training epochs. The solid lines show the mean AUC_Train (blue) and AUC_Val (green) across 5 repeats of 4-fold cross-validation. The shaded regions denote the 95% confidence intervals.


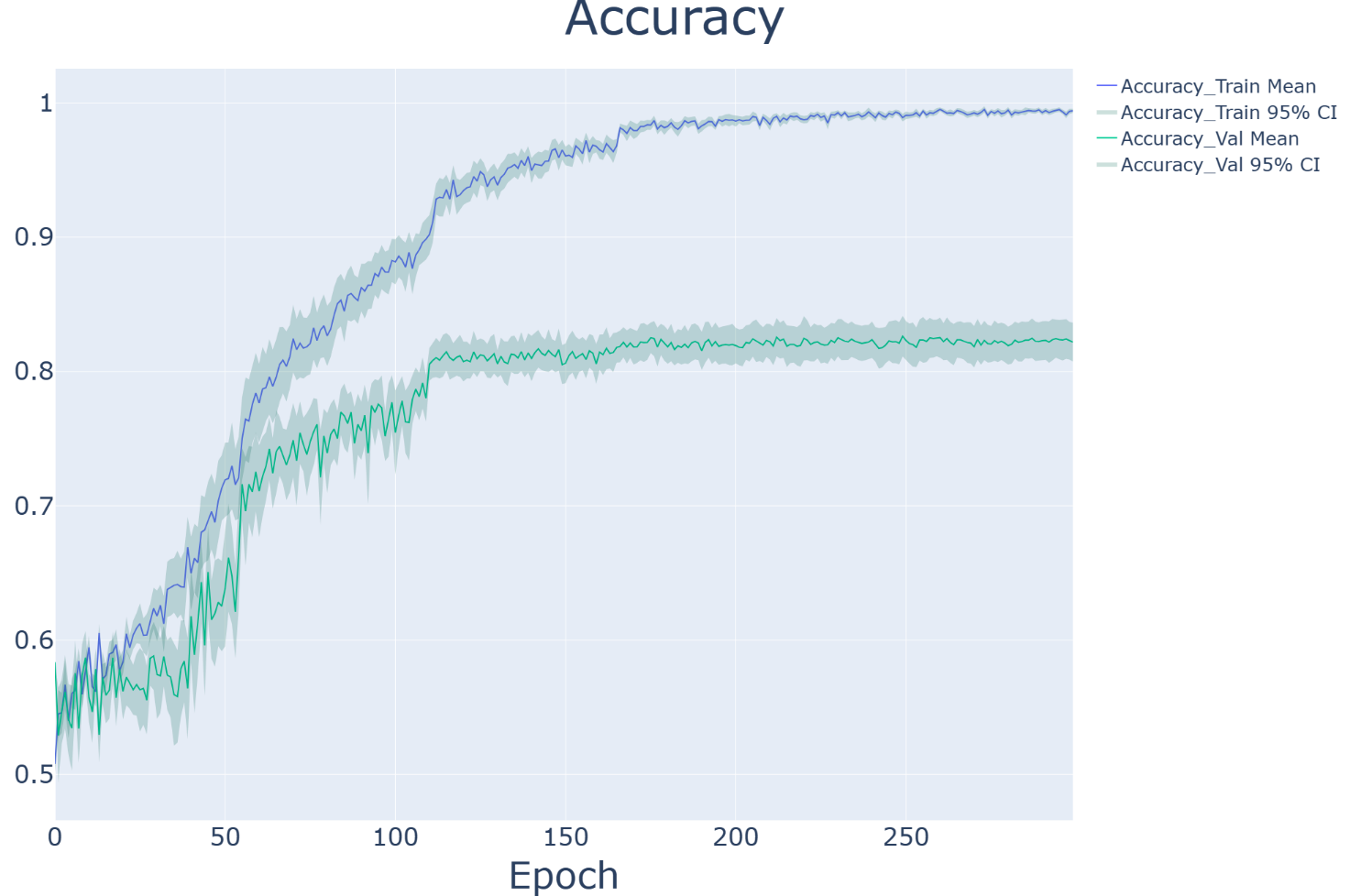


**Figure S4 Accuracy.** Accuracy on the training and validation sets over training epochs. The solid lines show the mean Accuracy_Train (blue) and Accuracy_Val (green) across 5 repeats of 4-fold cross-validation. The shaded regions denote the 95% confidence intervals.


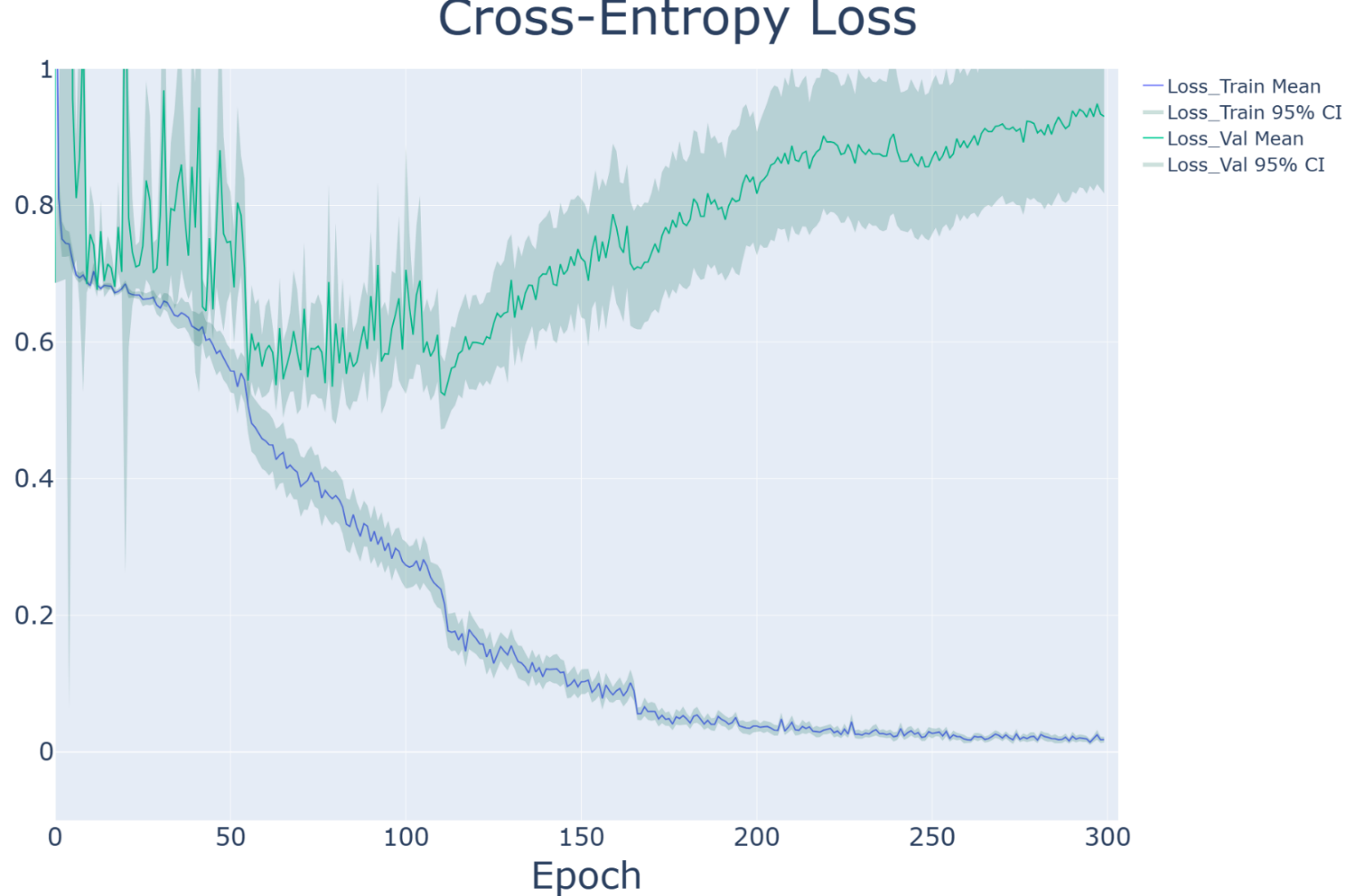


**Figure S5 Weighted Cross-Entropy Loss.** Weighted Cross-Entropy Loss on the training and validation sets over training epochs. The solid lines show the mean Loss_Train (blue) and Loss_Val (green) across 5 repeats of 4-fold cross-validation. The shaded regions denote the 95% confidence intervals.
